# Validation of α-Synuclein as a CSF Biomarker for Sporadic Creutzfeldt-Jakob Disease

**DOI:** 10.1007/s12035-017-0479-5

**Published:** 2017-03-21

**Authors:** Franc Llorens, Niels Kruse, André Karch, Matthias Schmitz, Saima Zafar, Nadine Gotzmann, Ting Sun, Silja Köchy, Tobias Knipper, Maria Cramm, Ewa Golanska, Beata Sikorska, Pawel P. Liberski, Raquel Sánchez-Valle, Andre Fischer, Brit Mollenhauer, Inga Zerr

**Affiliations:** 1 Clinical Dementia Center, Department of Neurology, University Medical Center Göttingen, Robert Koch Stasse 40, 37075 Göttingen, Germany; 2 German Center for Neurodegenerative Diseases (DZNE), Site Göttingen, Robert Koch Stasse 40, 37075 Göttingen, Germany; 3 Institute for Neuropathology, University Medical Center Göttingen, Göttingen, Germany; 4 Department of Epidemiology, Helmholtz Centre for Infection Research, Braunschweig, Germany; 5 Department of Molecular Pathology and Neuropathology, Medical University of Lodz, Lodz, Poland; 6 Creutzfeldt-Jakob disease unit. Alzheimer’s disease and other cognitive disorders unit. Hospital Clínic, Institut d’Investigacions Biomèdiques August Pi i Sunyer, Barcelona, Spain; 7 Paracelsus-Elena Klinik, Center for Parkinsonism and Movement Disorders, Kassel, Germany; 8 Department of Neurosurgery, University Medical Center Göttingen, Göttingen, Germany

**Keywords:** Sporadic Creutzfeldt-Jakob, α-Synuclein, Cerebrospinal fluid, Biomarkers, Electrochemiluminescence, ELISA

## Abstract

The analysis of cerebrospinal fluid (CSF) biomarkers gains importance in the differential diagnosis of prion diseases. However, no single diagnostic tool or combination of them can unequivocally confirm prion disease diagnosis. Electrochemiluminescence (ECL)-based immunoassays have demonstrated to achieve high diagnostic accuracy in a variety of sample types due to their high sensitivity and dynamic range. Quantification of CSF α-synuclein (a-syn) by an *in-house* ECL-based ELISA assay has been recently reported as an excellent approach for the diagnosis of sporadic Creutzfeldt-Jakob disease (sCJD), the most prevalent form of human prion disease. In the present study, we validated a commercially available ECL-based a-syn ELISA platform as a diagnostic test for correct classification of sCJD cases. CSF a-syn was analysed in 203 sCJD cases with definite diagnosis and in 445 non-CJD cases. We investigated reproducibility and stability of CSF a-syn and made recommendations for its analysis in the sCJD diagnostic workup. A sensitivity of 98% and a specificity of 97% were achieved when using an optimal cut-off of 820 pg/mL a-syn. Moreover, we were able to show a negative correlation between a-syn levels and disease duration suggesting that CSF a-syn may be a good prognostic marker for sCJD patients. The present study validates the use of a-syn as a CSF biomarker of sCJD and establishes the clinical and pre-analytical parameters for its use in differential diagnosis in clinical routine. Additionally, the current test presents some advantages compared to other diagnostic approaches: it is fast, economic, requires minimal amount of CSF and a-syn levels are stable along disease progression.

**Electronic supplementary material:**

The online version of this article (doi:10.1007/s12035-017-0479-5) contains supplementary material, which is available to authorized users.

## Introduction

Creutzfeldt-Jakob disease (CJD) is the most common human prion disease characterised by the accumulation of pathological misfolded prion protein (PrPSc) in the central nervous tissue. Most patients present an unknown aetiology being classified as sporadic Creutzfeldt-Jakob disease (sCJD), which is clinically characterised by the presence of dementia and ataxia [1, 2]. sCJD affects 1 to 2 per 1,000,000 individuals per year [3], and it is invariably fatal, usually within 1 year from disease onset [1]. Therefore, biomarkers able to discriminate sCJD from other neurological and neurodegenerative conditions with similar clinical presentation are required [4, 5]. This is of special importance in the differential diagnostics from alternative forms of rapidly progressive dementias with available treatment to generate an appropriate therapeutic intervention before pathological changes spread throughout the brain [6]. Established CSF biomarkers for sCJD tested in clinical workup in suspected cases are 14-3-3 and total tau (tau). A major limitation of these biomarkers results from the occurrence of their increased levels in acute neurologic disorders and neurodegenerative diseases [7–13]. The clinical diagnosis of sCJD is also supported by the real-time quaking-induced conversion (RT-QuIC) protein aggregation assay, which exhibit a high specificity, and sensitivity similar to the best surrogate biomarkers in detecting sCJD cases [14–17]. However, RT-QuIC presents increased costs, and less inter-laboratory standardisation data is available compared to classical approaches, preventing its widespread implementation in clinical routine.

In this context, electrochemiluminescence-based systems have demonstrated high accuracy, reproducibility, recovery rates and broad dynamic range in the detection of several biomarkers in biological fluids [18, 19]. Besides its methodological upgrade compared to classical colorimetric platforms, the analytical and clinical performance of electrochemiluminescence technology in the cerebrospinal fluid (CSF) has not been explored in detail in the differential diagnosis of neurodegenerative dementias.

The detection of elevated α-synuclein (a-syn) in the CSF of sCJD cases using colorimetric assays was previously reported [20, 21]. However, the differences between sCJD and control groups were rather limited, and the accuracy of the tests was low. Recently, we reported that the use of an *in-house* a-syn electrochemiluminescence-based enzyme-linked immunosorbent assay (ELISA) platform presented high accuracy in the discrimination of sCJD cases [19, 22]. However, the implementation of CSF a-syn measurements in diagnostic routine of sCJD would get benefit of the validation and establishment of diagnostic accuracy in a commercially available platform, facilitating its technical implementation and inter-laboratory standardisation.

In the present study, we analysed a commercial electrochemiluminescence-based human a-syn ELISA kit as a novel test method in sCJD diagnosis. The present test allowed discriminating non-CJD from sCJD cases with high diagnostic accuracy. Importantly, data were collected from three independent cohorts of sCJD autopsy-confirmed cases. Since standard protocols and sample handling have become more important in routine diagnostic, recommendations for the CSF a-syn diagnostic of sCJD are reported based on the study of the influence of pre-analytical and analytical parameters.

## Methods

### Subjects

The study included a total of 648 patients recruited at the National Reference Center for Transmissible Spongiform Encephalopathies of the Department of Neurology and Neuropathology at the University Medical Center of Gottingen, Germany (cohort 1), at Polish neurologic and psychiatric hospital departments (cohort 2) and at the Unit of Bio-diagnostic of CJD and other prion diseases at the Hospital Clinical of Barcelona, Spain (cohort 3). All patients with sCJD were classified as definite cases by neuropathological examination according to diagnostic consensus criteria [23]. Non-CJD cases were composed of patients referred to the same centres where prion disease diagnosis was excluded according to clinical criteria or autopsy. Non-CJD cases included the following: neurologically healthy patients (no neurological clinical diagnosis and normal neuro-physicological assessment) (*n* = 41) and patients suffering from neurological diseases (*n* = 149) or neurodegenerative conditions and cognitive impairment/dementia (*n* = 255). Neurological diseases included psychiatric disorders (psychosis, bipolar disorder, depression and schizophrenia), ischemic stroke, epilepsy, autoimmune diseases, meningitis, alcohol abuse, headache, vertigo, pain syndromes, acute hypoxia, encephalopathy, cerebral vasculitis, normal pressure hydrocephalus and alternative neurologic conditions. Neurodegenerative disease included Parkinson’s disease, dementia with Lewy bodies, multiple system atrophy, Alzheimer’s disease, frontotemporal dementia, Huntington’s disease, vascular dementia, corticobasal degeneration, progressive supranuclear palsy and olivopontocerebellar atrophy.

CSF samples were tested for blood contamination using Hemastix strips (Siemens), and cases containing more than 25 erythrocytes/mm^3^ and/or haemoglobin contamination were intentionally excluded from this study in order to avoid possible false positive outcomes. Information about demographics and CSF biomarkers tau and 14-3-3 of the study population are supplied in Table 1.

**Table 1:** Demographic characteristics of the study population analysed and CSF prion biomarker data

a		
*Demographics*	*non-CJD*	*sCJD*
Number of cases	445	203
Age (mean ± SD (years))	66 ± 12	66 ± 9
Gender (w/m in %)	54/46	52/48
*sCJD biomarkers*		
14-3-3 (p─w─n in %)	12─2─84	91─2─7
tau (mean ± SD (pg/mL))	402 ± 376	5965 ± 4911
b		
α *-synuclein (pg/mL)*	*non-CJD*	*sCJD*
Mean	324	8906
Standard deviation	214	7790
Standard error	10	548
Minimum	70	513
25% percentile	200	3183
Median	259	6595
75% percentile	364	11,950
Maximum	1908	41,960
Lower 95% CI	304	7828
Upper 95% CI	344	9984

a. The total number of cases, age (mean ± SD in years) and gender distribution as well as tau (mean ± SD in pg/mL) and 14-3-3 semi-quantification (% of positive (p), weak/inconclusive (w) and negative (n)) is reported. b. CSF a-syn levels (in pg/mL) in non-CJD and sCJD cases. Mean values, standard deviation and standard error, percentile values and lower and upper 95% CI are indicated

### Statistical Methods

Mann-Whitney *U* tests were used to compare two groups of samples. For analysis of a-syn stability, Wilcoxon signed rank sum tests were applied. In order to assess the diagnostic accuracy of a-syn, receiver operating characteristic (ROC) curve analyses were carried out and areas under the curve (AUC) with 95% confidence intervals were calculated using GraphPad Prism 6.01. The best cut-off value was estimated based on the Youden index [24] derived from cohorts 2 and 3 and was then externally validated in cohort 1. To compare the sensitivities between biomarkers, the McNemar’s test was used. Spearman rank correlation was used to test association between biomarker levels. For codon 129 polymorphism comparison, cases were tested for normality, and Kruskal-Wallis test followed by Dunns post-test was applied. In order to determine the effect of a-syn levels on clinical outcome, the association between a-syn levels and total disease duration (time between disease onset and death) was assessed using a fractional polynomial approach.

### CSF Analysis

Quantification of a-syn was performed using the electrochemiluminescence-based ELISA-based human a-syn kit from Meso Scale Discovery (MSD) (Catalogue No. K151TGD) following manufacturer’s instructions. Briefly, assay plates were blocked by adding 150 μl of Diluent 35 to each well. Plates were sealed and incubated at room temperature with shaking at 300 rpm for 1 h. Then, plates were washed three times with 150 μl per well of PBS-T (PBS supplemented with 0.05% Tween-20). Detection antibody (25 μl) and CSF (diluted 1:8) or calibrator (25 μl) was added to each well. Plates were sealed and incubated at room temperature with shaking at 700 rpm for 2 h. After washing as indicated above, 150 μl of 2× read buffer T was added to each well. Measurements were performed on a MSD Sector Imager 6000. Calibrators were run in duplicate to generate calibration curve.

Tau was quantified using the INNOTESThTAU-Ag (Fujirebio, Gent, Belgium) ELISA test. 14-3-3 was semi-quantitatively tested by using Western blot as previously described [8].

### Analysis of a-syn Stability

To evaluate the effects of CSF storage conditions (temperature and freezing/thawing cycles) on a-syn concentration, CSF samples from 8 sCJD patients were stored in polypropylene tubes at room temperature and 4 °C for 1, 3 and 6 days. In addition, CSF samples were subjected to 2, 4 and 8 repeated freezing and thawing cycles. a-syn concentrations at each time point or cycle were calculated as percent of control (time point zero), which was defined as 100%.

### Ethics

The study was conducted according to the revised Declaration of Helsinki and Good Clinical Practice guidelines and approved by local Ethics committees Goettingen (No. 9/6/08, 19/11/09, 18/8/15). Informed consent was given by all study participants or their legal next of kin.

## Results

### ELISA Test Performance in the Quantification of CSF a-syn Quantification

**Fig. 1:**
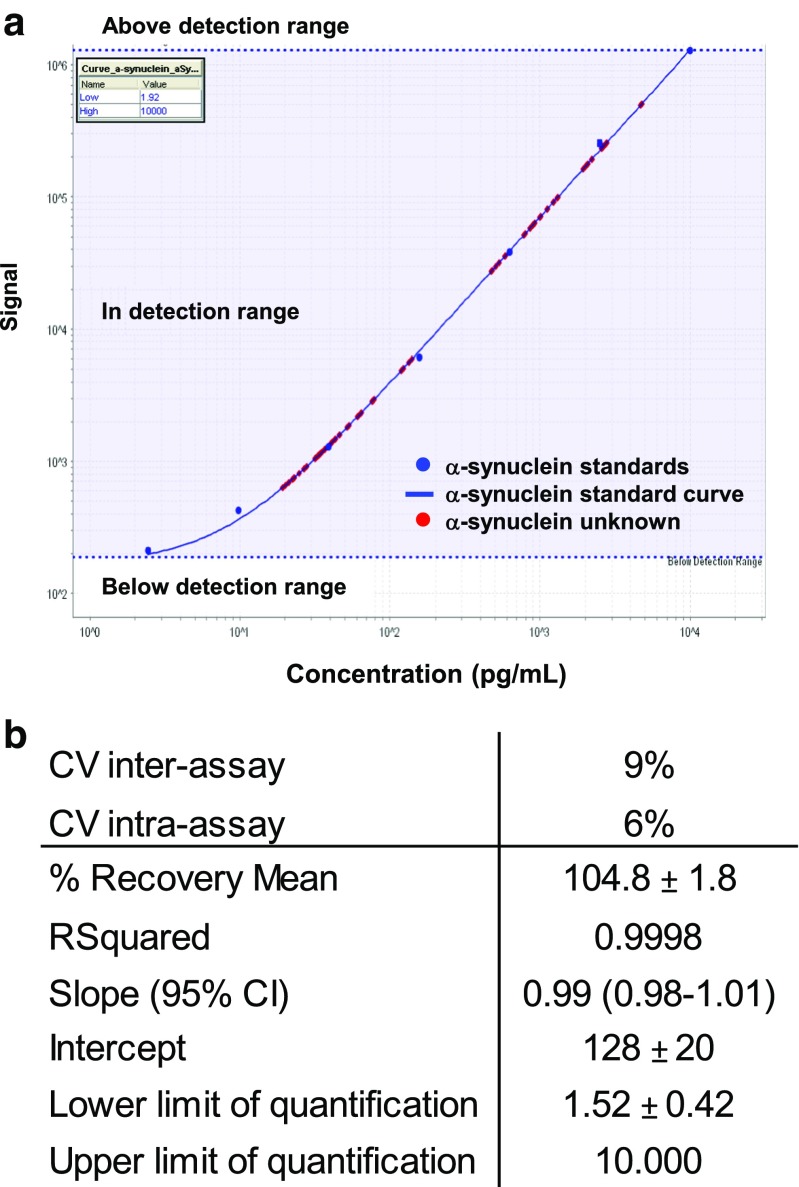
Quantification of CSF a-syn by the human ELISA kit from MSD. **a** Representative plot for quantification of CSF a-syn in samples from CJD and non-CJD cases. The dynamic range is indicated in *blue*. Calibrators are denoted as blue spots and unknown samples as red spots. CSF samples from diverse aetiology were in detection range using a 1:8 dilution. **b** Analytical and technical parameters derived from quantification of CSF a-syn with the human ELISA kit from MSD are shown

CSF a-syn levels were measured using the electrochemiluminescence-based human a-syn kit (Fig. 1a). The dynamic range of the assay was from 1.52 ± 0.42 to 10,000 pg/mL a-syn. The precision of the assay was validated by the low coefficients of variation (CV) measured in separate runs, samples, laboratories and analysers as suggested elsewhere [25]. In all cases, repeatability and within-lab/plate reproducibility were below 9% (Fig. 1b), validating precision and limits of quantification of the assay. Inter-lot and inter-laboratory variability were lower than 11 and 12%, respectively. Dilution linearity was assessed in serial dilutions of CSF to avoid hook effect. Selected CSF dilution (1:8) permitted the lineal quantification of the lowest and highest a-syn levels without the need to analyse multiple sample dilutions.

### Diagnostic Accuracy of CSF a-syn as sCJD Biomarker

**Fig. 2:**
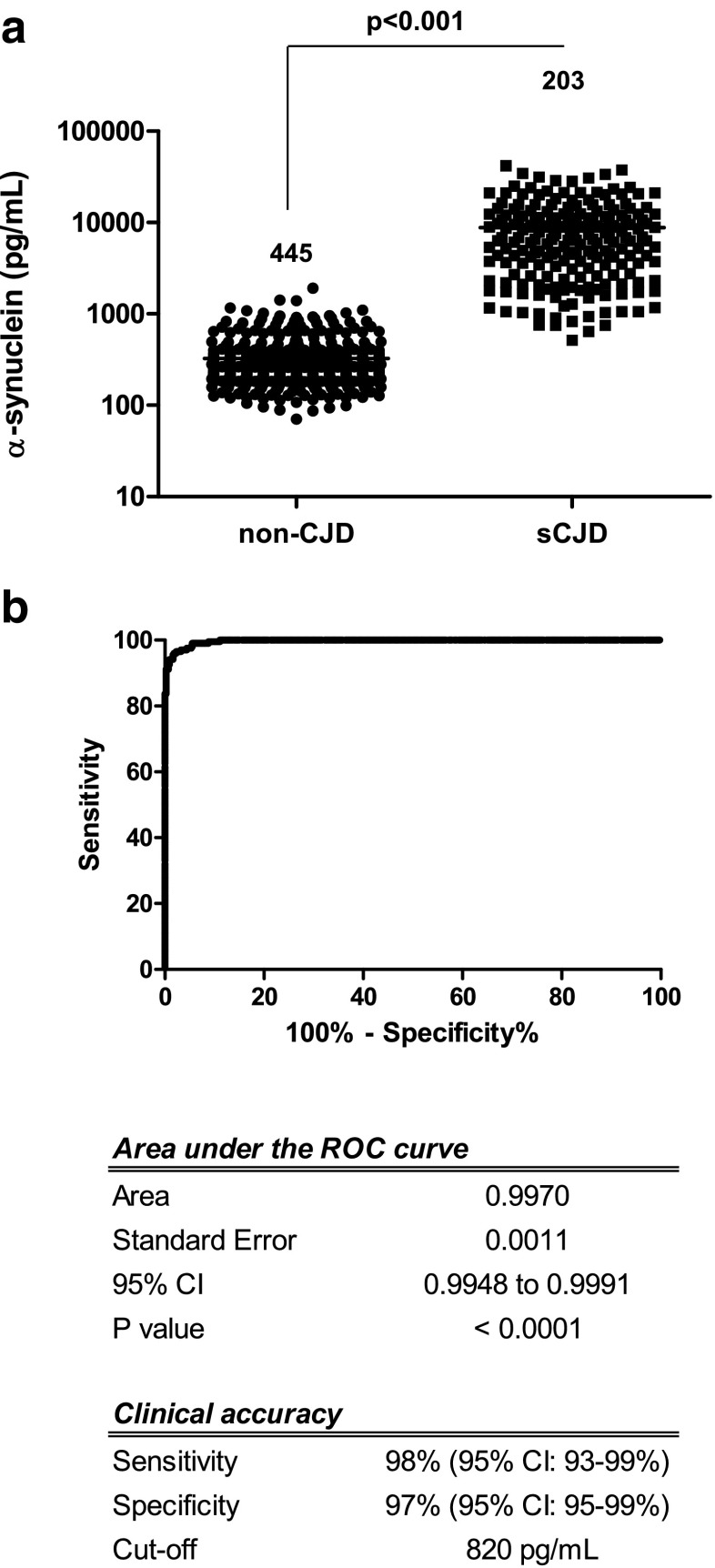
Diagnostic accuracy of CSF a-syn levels as sCJD biomarker. **a** CSF a-syn levels in non-sCJD and sCJD. Statistically significant differences were detected between non-CJD and sCJD cases (*p* < 0.001). **b** ROC curve for a-syn in the comparative analysis between non-sCJD cases and sCJD cases. Sensitivity and specificity, receiver operating characteristic (ROC) curves and derived area under the curve (AUC) were calculated. Based on Youden Index, an optimal cut-off of 820 pg/mL a-syn and a sensitivity of 98% and specificity of 97% were achieved for the discrimination of sCJD from non-CJD cases

Our study cohort included a total of 445 non-CJD and 203 sCJD cases. CSF a-syn showed an excellent accuracy in discriminating non-CJD from sCJD cases (*p* < 0.001 and AUC = 0.997, 95% CI: 0.995 to 0.999) (Fig. 2a, b). Mean a-syn values were 324 ± 214 in non-CJD and 8906 ± 7790 in sCJD (Table 1b). Using a cut-off of 820 pg/mL of a-syn (optimal level identified by Youden index in the training cohorts), a sensitivity of 98% (95% CI: 93–99%) and a specificity of 97% (95% CI: 95–99%) was achieved in the validation cohort (Fig. 2b). Since high sensitivities are reported for CSF 14-3-3 and tau tests in the detection of sCJD cases [7–13, 26, 27] (Supplementary Table 2), we aimed to compare the performance of the three biomarkers in the same population. 14-3-3 and tau from cohort 1 (*n* = 128) presented sensitivities of 91 and 92%, respectively, in detecting sCJD cases, which was significantly lower than those obtained for CSF a-syn (98%). Neither a specific diagnostic group nor a correlation between a-syn and tau/14-3-3 profile was detected in the non-CJD cases tested positive for a-syn. Comparison of sensitivities in cohort 1 between the three biomarkers revealed statistical differences between a-syn vs tau (*p* = 0.035) and a-syn vs 14-3-3 (*p* = 0.021) comparisons, but not in tau vs 14-3-3 (*p* = 0.763). Additionally, we found a positive correlation between CSF a-syn and tau levels (*p* < 0.001) in control subjects and in sCJD cases, in agreement with the observations from our previous study [22]. Next, we investigated the role of *PRNP* codon 129 polymorphism on CSF a-syn levels. Information about codon polymorphism was available for 182 cases (MM = 120, MV = 23 and VV = 34). As we previously reported [22], a-syn levels in MV cases (5391 ± 4780 pg/mL a-syn) were significantly lower than those detected in MM (9695 ± 7605 pg/mL a-syn) (*p* < 0.05). Mean values in MV cases were also lower than in VV (9941 ± 9733 pg/mL a-syn). However, these differences were not statistically significant.

### Influence of Timing in CSF Tests and of Disease Duration

**Fig. 3:**
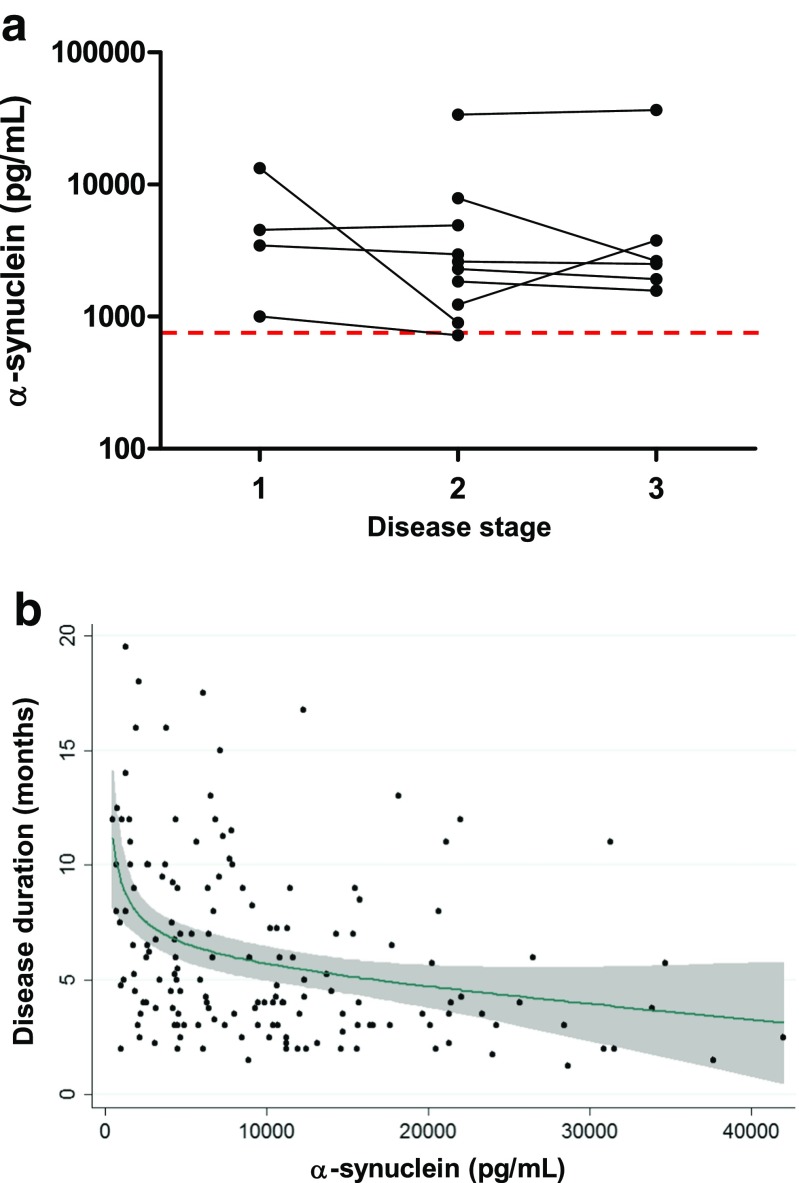
Influence of timing and disease duration in CSF a-syn levels. **a** CSF a-syn levels in serial LPs in sCJD cases at different stages of the disease. Samples were grouped in three categories according to whether they underwent LP in the first (time from disease onset to LP/total duration of the disease <0.33), second (0.33–0.66) or third (>0.66) stage of the disease. *Dashed line* indicates established cut-off for sCJD. **b** Association between CSF a-syn levels and disease duration (months) in sCJD patients analysed by a fractional polynomial linear regression approach (*p* < 0.001). Displayed is the functional form of the association with 95% confidence intervals. The expected duration of disease can be estimated by using the following equation: duration in months = 5.70 + 1.43*((a-syn (pg/mL)/10,000)^-0.5^–0.99)–0.57*((a-syn (pg/mL)/10,000)–1.02)

Next, we assessed the influence of time of CSF sampling on a-syn concentration in the diagnosis of sCJD. For this, samples from 10 sCJD patients for which CSF at different stages of disease was available were tested. Since disease duration in sCJD is highly variable, depending on demographic and genetic characteristics [2], the time interval between disease onset and lumbar puncture (LP) is not an appropriate estimate of the stage of disease when the LP was performed. Instead, we divided the time of LP to disease onset in each patient by the total duration of the disease. Then, samples were grouped in three categories according to whether they underwent LP in the first (time of LP to disease onset/total duration of the disease <0.33), second (0.33–0.66), or third (>0.66) stage of the disease, as previously reported [28]. No major alterations in CSF a-syn concentration were detected between different LPs in nine of the cases (Fig. 3a). One case presented a decrease in a-syn levels, most likely related to its rapid disease course (3 weeks from disease onset to death).

CSF a-syn levels were inversely associated with disease duration (*p* < 0.001) in the 185 cases where disease duration was known (Fig. 3b). This suggests a role for CSF a-syn as a prognostic sCJD marker. In this regard, our data indicate that when a-syn levels are higher than 20,000 pg/mL, disease duration was inevitably shorter than 1 year. By using fractional polynomial linear regression analysis, the expected duration of disease from the a-syn value can be estimated by using the following equation: duration in months = 5.70 + 1.43*((a-syn (pg/mL)/10,000)^−0.5^–0.99)–0.57*((a-syn (pg/mL)/10,000)–1.02).

### Effect of Multiple Freeze/Thaw Cycles and Defined Storage Conditions

**Fig. 4:**
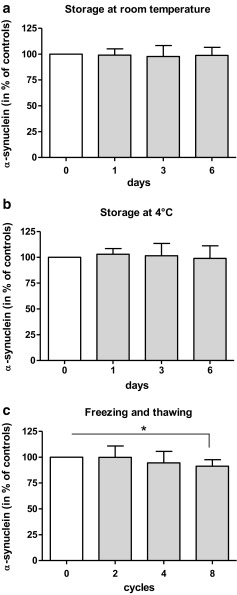
Effect of short- and long-term storage on CSF a-syn concentration in sCJD patients. CSF a-syn levels were determined as indicated in “Material and Methods”. CSF a-syn concentration is shown relative to the reference sample (time point 0), which was set as 100%. The concentration of CSF a-syn from 8 different sCJD patients (*n* = 8) was determined **a** before and after storage at room temperature (0 to 6 days), **b** storage at 4 °C (0 to 6 days) and **c** after repeated freezing and thawing cycles (0 to 8 cycles). A significant loss of CSF a-syn level could be observed in samples subjected to 8 freezing-thawing cycles. *Error bars* represent standard deviations (SD)

Storage of CSF at room temperature or at 4 °C for up to 6 days had no effect on a-syn concentration (Fig. 4a, b). CSF a-syn was stable for up to four freezing/thawing cycles while a decrease of 9.7 ± 6.7% (*p* < 0.05) was detected after 8 cycles (Fig. 4c). Additionally, CSF storage for 12 months at −80 °C did not alter the levels of a-syn in a subset of 3 non-CJD and 3 sCJD cases (data not shown).

## Discussion

CSF a-syn quantification by an *in-house* electrochemiluminescence-based ELISA platform has been recently reported as a useful biomarker in the discrimination of sCJD cases from a broad range of neurological and neurodegenerative disorders [22]. However, implementation of the assay in clinical diagnostics requires the following: (i) the validation and standardisation of a commercially available kit, along with definition of diagnostic accuracy and cut-off values, and (ii) investigation of relevant pre-analytical parameters to be considered for sample collection and management. In the present study, the human a-syn kit from Meso Scale Discovery® has been validated as a diagnostic test for sCJD. Clinical accuracy has been established, and pre-analytical parameters for appropriate sample handling are reported. The ELISA kit showed high reproducibility (intra- and inter-assays <9%) and sensitivity and a broad dynamic range. The wide dynamic range of the detection system (4 logs) allowed the quantification of a-syn levels without the need of multiple sample dilutions. Because of the high sensitivity of the technology, lower amounts of CSF are required compared to other approaches. Other advantages of the present system include its low background since stimulation method (electricity) is coupled to the signal (light) allowing only labels near the electrode surface to be detected.

In the study of the accuracy of a-syn levels as sCJD biomarker, we analysed one of the largest studied cohorts with definite prion disease. At the optimal cut-off of 820 pg/mL a-syn, we obtained a specificity of 98% and a sensitivity of 97% in the validation cohort. Importantly, biomarker cut-off was externally validated in an independent cohort from a different country, underlining the relevance of the findings.

Overall, a-syn test shows an optimal diagnostic accuracy in the specific detection of sCJD cases, with clinical parameters in the range of the best currently available prion biomarkers (Supplementary Table 2) such as p-tau/tau ratio [29] and the newly implemented real-time quaking-induced conversion RT-QuIC [15]. Additionally, the present ELISA tests displayed a similar analytical performance as previously reported for an *in-house* electrochemiluminescence-based ELISA platform [22], reinforcing the robustness of the method.

Several CSF biomarkers are currently used as valuable tools for the diagnosis of sCJD. While most of them are surrogate disease markers (14-3-3, tau, S100B, NSE) [7], RT-QuIC directly measures the amount of pathogenic prion protein able to induce the conversion of recombinant PrP molecules in a fluorimetric seeding assay [15–17]. Surrogate markers with highest sensitivity such as 14-3-3 and tau present lower specificity when tested on several neurological conditions [7, 29, 30]. Indeed, positive 14-3-3 signal can be detected in the CSF of various neurological disorders such as acute stroke, meningoencephalitis, and subarachnoidal haemorrhage [31, 32], while elevated tau is a common hallmark in several types of neurodegenerative dementias (Alzheimer’s disease, mild cognitive impairment, vascular dementia, and fronto-temporal dementia) [29, 33] and in acute ischemic events [34]. On the contrary, elevated a-syn is not detectable in any group of neurological and neurodegenerative disorders as reported before [22], and in the present study, enhancing the specificity of the test over other biomarker approaches. In this regard, RT-QuIC overcomes the handicap of surrogate biomarkers regarding specificity; however, sensitivity is still not optimal as shown in several other studies (85–96%) [16]. Furthermore, a-syn measurement is faster (3.5 h) than for first and second generation RT-QuIC assays (<2 days and 4–14 h, respectively) [15, 17]. Regarding pre-analytical conditions, some inconsistencies are reported in the bibliography. Regarding pre-analytical conditions, some inconsistencies are reported in the bibliography. While CSF a-syn levels were found to be decreased after 4 days at 4 °C and 6 freeze/thaw cycles [35], Kruse et al., in agreement with our observations, showed a great stability on a-syn levels on a broad range of pre-analytical conditions, including freeze/thawing cycles and temperature storage conditions [36]. A limitation of CSF a-syn quantification is the increased abundance of a-syn in peripheral blood [37]; thus, CSF samples must be tested for blood contamination in relation to this test. Indeed, presence of blood contamination is usually tested in prion diagnosis routine due to its influence in other CSF biomarkers such as 14-3-3 and RT-QuIC [17, 27]. Although new generation ELISA platforms are not yet implemented in most clinical routine laboratories, their reported technical advantages over classical colorimetric systems and the possibility to simultaneously test different analytes in a single run will help to increase its widespread implementation in dementia diagnostic work up.

sCJD disease duration varies depending on demographic and genetic factors from several weeks to 3 years [3], with an average disease duration of 5 months, and only 14% of the patients surviving longer than 1 year (Creutzfeldt-Jakob Disease International Surveillance Network, http://www.eurocjd.ed.ac.uk). Here, we also demonstrated that CSF a-syn levels are stable along disease duration as reported in a group of sCJD cases ranging from the lowest to the highest a-syn levels for which serial LPs were available. Altogether, our study supports the use of CSF a-syn quantification in diagnostic routine as a first robust, cheap and fast discriminatory assay to detect sCJD cases, which could be further validated by means of RT-QuIC test to achieve a defined pre-mortem diagnosis.

We summarised the outcome of our study and prepared a recommendation table for pre-analytical sample handling and storage to improve standardisation of CSF a-syn quantification for the diagnosis of sCJD patients in clinical routine (Table 2). See also recommendations for sample handling published recently [35].

**Table 2:** Recommendations for standardisation and pre-analytical treatment of CSF samples for the analysis of a-syn in sCJD diagnostics

1 Tube selection	Polypropylene tubes
2. CSF volume	0.5–1.5 mL
3. Shipping time	Up to 6 days at 4 °C or room temperature
4. After arrival	Specimen macroscopically clear
	Assessment for blood contamination inspection
	supplemented with hemasticks or routine
	microscopic quantification
	Centrifugation at 1500 xg for 10 min
	Freeze at −80 °C until test is performed
5. Freeze/thaw	Stable for at least 6 cycles
6. Long-term storage	Stable for at least 12 months at −80 °C

A potential further application of CSF a-syn quantification is its use as prognostic marker, since disease duration showed a negative correlation with a-syn levels, comparable to CSF tau [38]. In this regard, a valuable outcome of our work is the presentation of an equation to estimate the expected duration of disease from the a-syn value. Indeed, identification of genetic, demographic and biochemical predictors of survival time is of crucial interest, not only for diagnosis and counselling, but also for the correct assessment and evaluation of an eventual therapeutic intervention.

*a-syn*, α-synuclein; *AUC*, area under the curve; *CJD*, Creutzfeldt-Jakob disease; *ECL*, Electrochemiluminescence; ELISA, enzyme-linked immunosorbent assay; *LP*, lumbar puncture; *CSF*, cerebrospinal fluid; *PrPSc*, pathological misfolded prion protein; *ROC*, receiver operating characteristic; *RT-QuIC*, real-time quaking-induced conversion; *sCJD*, sporadic Creutzfeldt-Jakob disease; SD, standard deviation.

## Electronic supplementary material

Supplementary Table 1 Number of cases and a-syn values stratified for the three different cohorts analysed in the present study (A) and stratification of controls according to differential diagnosis (B). (PPTX 57 kb)

Supplementary Table 2 Comparison of CSF sCJD biomarkers according to literature. Biomarker outcome, methodology and clinical accuracy values (sensitivity and specificity) expressed in ranges, when more than one study is cited, are reported. (PPTX 69 kb)
