# Supplementary figures and images for: Validation of α-Synuclein as a CSF Biomarker for Sporadic Creutzfeldt-Jakob Disease
Source: Mol Neurobiol. 2017 Mar 21;55(3):2249–57. doi: 10.1007/s12035-017-0479-5 (PMC5840235; doi:10.1007/s12035-017-0479-5)

## Slide 1
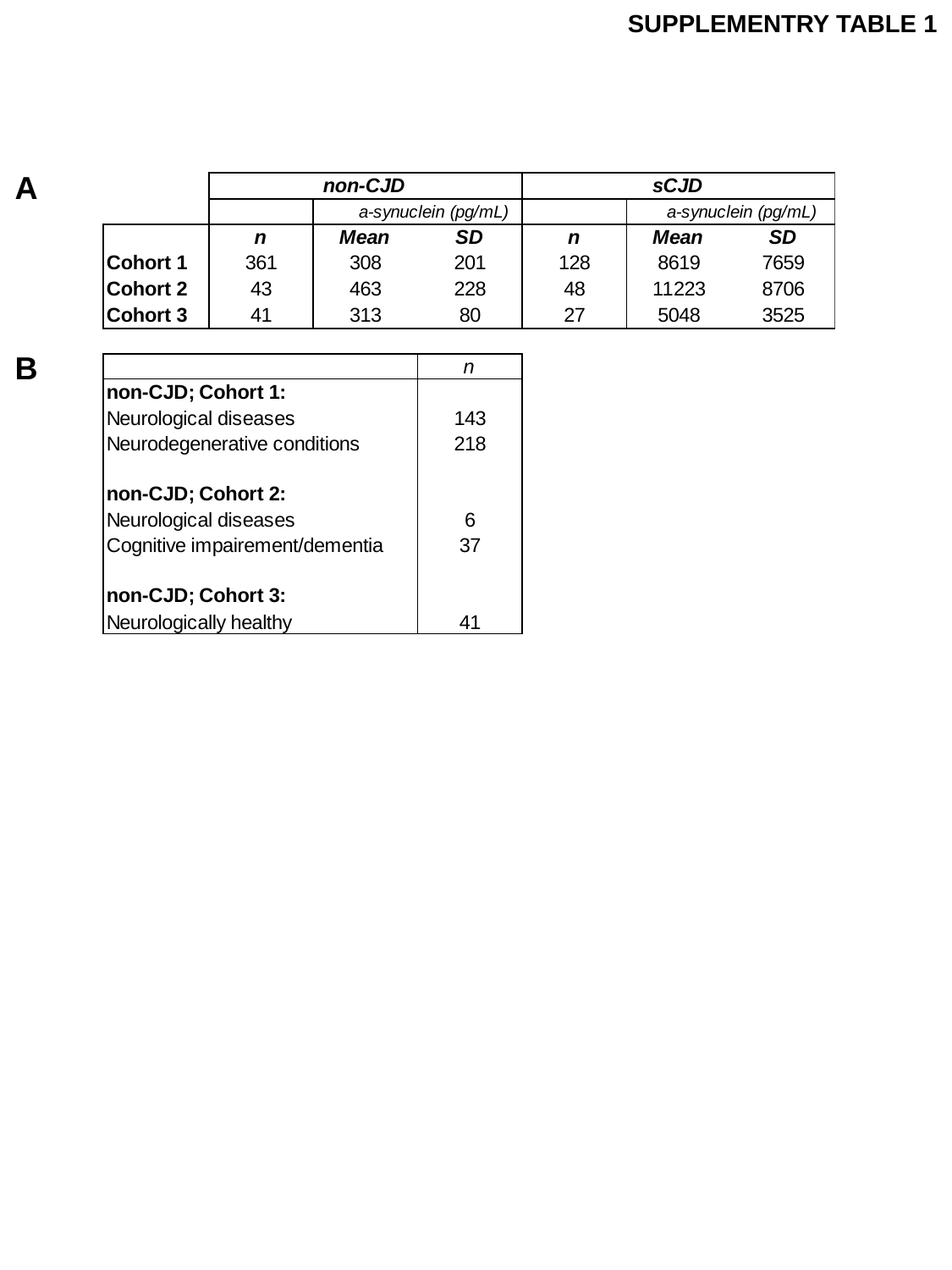

SUPPLEMENTRY TABLE 1
A
B

Supplement: Supplementary file 1 — Number of cases and a-syn values stratified for the three different cohorts analysed in the present study (A) and stratification of controls according to differential diagnosis (B). (PPTX 57 kb) [file 12035_2017_479_MOESM1_ESM.pptx]

## Slide 1
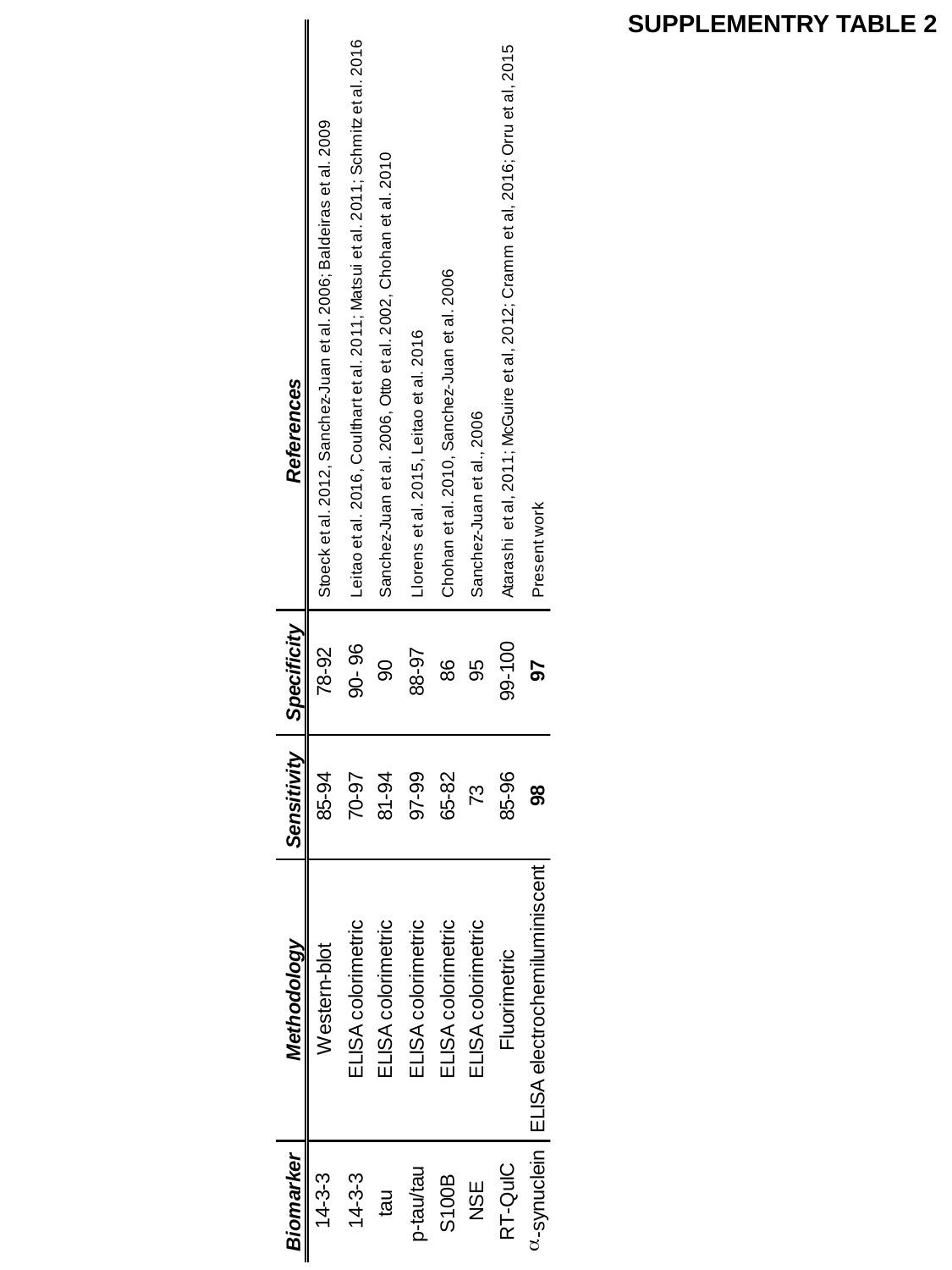

SUPPLEMENTRY TABLE 2

Supplement: Supplementary file 2 — Comparison of CSF sCJD biomarkers according to literature. Biomarker outcome, methodology and clinical accuracy values (sensitivity and specificity) expressed in ranges, when more than one study is cited, are reported. (PPTX 69 kb) [file 12035_2017_479_MOESM2_ESM.pptx]
